# Effect of Different Fat and *Moringa oleifera* Leaf Meal (MOLM) Inclusion Levels on Proximate Composition, Fatty Acid Profile, and Lipid Oxidation of Chicken Droëwors

**DOI:** 10.1155/2022/6736935

**Published:** 2022-09-06

**Authors:** Nelisiwe Tembela, Felicitas Esnart Mukumbo, Arno Hugo, Ishmael Festus Jaja

**Affiliations:** ^1^Department of Livestock and Pasture Sciences, Faculty of Science and Agriculture University of Fort Hare, P/Bag X1314, Alice 5700, South Africa; ^2^Rwanda Institute for Conservation Agriculture, Kagasa-Rweru Road, Gashora/Rweru Districts, Bugesera, Rwanda; ^3^Department of Microbial Biochemical and Food Biotechnology, University of Free State, P.O. Box 339, Bloemfontein 9300, South Africa; ^4^Department of Agriculture and Animal Health, University of South Africa, Johannesburg, South Africa

## Abstract

We present the first report on the effect of graded levels of *Moringa oleifera* leaf meal (MOLM) (0, 0.25, and 0.5%) and fat (0, 10, and 15%) on fatty acid profile, lipid oxidation, and proximate composition of chicken droëwors. On triplicate samples of all treatments, proximate analysis was done, the total lipid was quantitatively extracted using chloroform and methanol in a ratio of 2 : 1, fatty acid profiles were determined, and thiobarbituric acid reactive substances (TBARS) were measured. The present study showed that droëwors manufactured with 0% fat inclusion had less fat and more protein than those made with 10% and 15% fat. All treatments contained a greater percentage of C18:1c9 (oleic) (30.95 to 32.65%) acid than other fatty acids and a higher proportion of unsaturated fatty acids than saturated. T9 (15% fat, 0.5% MOLM) had significantly (*P* < 0.05) higher PUFAs than T1 (0% fat, 0% MOLM) and T4 (10% fat, 0% MOLM). Treatments with 0.5% MOLM had significantly lower TBARS values after drying (0.01-0.07 mg MDA/kg) than treatments with 0% and 2.5% MOLM (0.05–0.15 mg MDA/kg). Therefore, MOLM inclusion at 0.25 and 0.5% effectively decreased TBARS of chicken droëwors with up to 15% fat inclusion after 72 h of drying and 168 h of storage and is a potentially good source of natural antioxidants for this traditional dried sausage product.

## 1. Introduction

Droëwors is a dried traditional South African salted sausage product typically made using beef or a variety of game meats [1]. Chicken meat is recognized to have high polyunsaturated fatty acid (PUFA) content, making it susceptible to lipid oxidation [2]. Meats with high PUFA content are generally not often considered for processing dried meat products such as droëwors, as the grinding and drying processes accelerate the rate of lipid oxidation even further [3]. Chicken meat oxidation results in quality deterioration decreased shelf life and increased off-flavours [4], negatively impacting the product's sensory and nutritional value and subsequently affecting consumers' acceptance of the product. Meat oxidation can also alter meat's chemical composition, increasing carcinogenic substances' levels. Consumers reject meat products that have undergone oxidation [5]. Therefore, limiting the lipid oxidation progression in meat and processed meat by-products is necessary. The stoppage of the lipid oxidation progression will enhance the potential for producing droëwors using chicken meat. Due to several health concerns associated with synthetic antioxidants [6], an increasingly popular mechanism to deter lipid oxidation is adding natural antioxidants to delay the process [7].

*Moringa oleifera* grows naturally in various countries. It has multiple names, such as the drumstick tree, ben oil, and horseradish [8]. This plant is common because its seeds flowers and leaves are used in human nutrition and herbal medicine [9]. Originally, the plant was incorporated in animal feeds and as an ingredient in traditional medicines. However, the *Moringa oleifera* plant possesses numerous multifunctional applications in human nutrition and animal nutrition and in the processing of animal food products [10]. *Moringa oleifera* leaves are high in nutrients such as high potassium, iron, protein, and vitamin C [11]. This plant could be eaten fresh or dried and stored as a powder without refrigeration with no nutritional loss after numerous months of storage [12]. *Moringa oleifera* is a powerful natural antioxidant since it contains flavonoids, tocopherols, vitamin C, and other phenolic compounds [9].

Consumers are more careful about the food quality, especially the nutritional attributes. Fatty acid profiles are among the dietary characteristics that consumers are concerned about [13]. In meat products, the fatty acid profiles can be influenced by several factors, including animal species, diet, and the level of fat incorporation during processing. Therefore, the current study added *Moringa oleifera* leaf meal (MOLM) to chicken droëwors with different fat levels to prevent lipid oxidation.

## 2. Materials and Methods

### 2.1. Ethical Consideration

Ethical clearance (Ethical Clearance Number: MUC561STEM01) was obtained from the University of Fort Hare Research Ethics Committee before the research commenced.

### 2.2. Production of Chicken Droëwors and Addition of MOLM

Lean chicken meat, chicken fat, dehydrated natural sheep casings (22 mm diameter, Freddy Hirsch), salt, and ground black pepper were purchased for droëwors preparation. The lean meat and fat were trimmed into 5 × 5 cm cubes and were divided into nine (9) batches and nine (9) treatments. The treatments were prepared with different combinations of fat and MOLM (Table 1). The chicken droëwors were prepared by mincing each batch of lean meat and fat through a 5 mm grinder, thoroughly incorporating salt (2%), pepper (0.5%), and MOLM according to the treatment levels described in Table 1. The batches were stuffed separately into natural sheep casings and were hung vertically in a drying chamber at 30°C and 40% relative humidity for 72 hours.

### 2.3. Proximate Analysis

After drying (72 h), triplicate samples (±50 g each) of each treatment were analysed for proximate composition. Moisture (Method 934.01) and ash (Method 924.05) contents of the dried droëwors were determined according to the methods defined in [14]. The protein content was determined according to [14] (Method 992.15), and fat content was determined using the chloroform/methanol (2 : 1) fat extraction method illustrated in the literature [15]. All analyses were conducted in duplicate.

### 2.4. Determination of Fatty Acids

After drying (72 h), triplicate samples (±50 g each) of each treatment were analysed for fatty acid composition. Total lipid from dry sausage was quantitatively extracted, as described in the literature, using chloroform and methanol in a ratio of 2 : 1 [16]. Antioxidant butylated hydroxytoluene was added at a concentration of 0.001% to the chloroform : methanol mixture. A rotary evaporator was used to dry the fat extracts under a vacuum. The extracts were dried overnight in a vacuum oven at 50°C, using phosphorus pentoxide as a moisture adsorbent. Total extractable fat from dry sausage was determined gravimetrically from the extracted fat and expressed as percent fat (*w*/*w*) per 100 g tissue.

The fat-free dry matter (FFDM) content was determined by weighing the residue on a preweighed filter paper used for Folch extraction after drying. The FFDM was expressed as % FFDM (*w*/*w*) per 100 g tissue by determining the difference in weight. The muscle moisture content and methanol BF-3 were determined by subtraction (100% − %lipid − %FFDM) and expressed as % moisture (*w*/*w*) per 100 g tissue. The extracted fat from dry sausage was stored in a poly-top (glass vial, with push-in top) under a blanket of nitrogen and frozen at -20°C pending fatty acid analyses.

A lipid aliquot (20 mg) of dry sausage lipid was transferred into a Teflon-lined screw-top test tube using a disposable glass Pasteur pipette. Fatty acids were trans-esterified to form methyl esters using 0.5 N NaOH in methanol and 14% boron trifluoride in methanol [17]. FAMEs from dry sausage were quantified using a Varian 430 flame ionization GC, with a fused silica capillary column, Chrompack CPSIL 88 (100 m length, 0.25 mm ID, and 0.2 *μ*m film thicknesses). The analysis was performed using an initial isothermic period (40°C for 2 minutes). Thereafter, the temperature was increased at a rate of 4°C/minute to 230°C. Finally, an isothermic period of 230°C for 10 minutes followed. FAMEs n-hexane (1 *μ*L) were injected into the column using a Varian CP 8400 Autosampler. The injection port and detector were both maintained at 250°C. At 45 psi, hydrogen functioned as the carrier gas, while nitrogen was employed as the makeup gas. Galaxy Chromatography Software recorded the chromatograms.

Fatty acid methyl ester samples were identified by comparing FAME peaks' retention times from samples with those of standards obtained from Supelco (Supelco 37 Component Fame Mix 47885-U, Sigma-Aldrich Aston Manor, Pretoria, South Africa). All other reagents and solvents were of analytical grade and obtained from Merck Chemicals (Pty Ltd, Halfway House, Johannesburg, South Africa). Fatty acids were expressed as the proportion of each individual fatty acid to the total of all fatty acids present in the sample. The following fatty acid combinations were calculated: omega-3 (n-3) fatty acids, omega-6 (n-6) fatty acids, total saturated fatty acids (SFA), total monounsaturated fatty acids (MUFA), polyunsaturated fatty acids (PUFA), PUFA/SFA ratio (P/S), and n-6/n-3 ratio.

### 2.5. Lipid Oxidation

For analysis of the evolution of lipid oxidation, sampling of chicken droëwors was implemented in triplicate (±50 g each) from each batch at 0, 0.25, 0.5, and 72 h during drying. The droëwors were stored, unpackaged, and uncovered in a ventilated room under ambient conditions and sampled after 7 days (168 h). The content of thiobarbituric acid reactive substances (TBARS), determined at 0, 0.25, 0.5, 72, and 168 hrs, was performed using the technique described in the literature [18]. Briefly, samples were homogenized with 12.5 mL of TCA (20% trichloroacetic acid in 1.6% metaphosphoric acid (HPO_3_)) and 12.5 mL distilled water for 180 sec using a Stomacher 400 Laboratory Blender (Seward Medical, London, UK). Slurries were filtered (0.45 *μ*m), and duplicate samples of filtrate (3 mL) were added to an equal volume of 0.02 M 2-thiobarbituric acid. An equal volume of distilled water was added to the third replicate to act as a turbidity blank for each sample. Samples were vortexed for 10 sec, incubated in a water bath at 70°C for 1 hour until pink colour development, and allowed to cool for 30 min, and the absorbance was recorded at 532 nm. TBARS were calculated using 1,1,3,3-tetraethoxypropane (TEP) as a standard. Results were expressed as mg of malonaldehyde (MDA) equivalents/kg of meat.

### 2.6. Data Analysis

The data on lipid oxidation, proximate composition, and fatty acid profile of chicken droëwors were analysed using the PROC GLM procedures of [19], and pairwise comparisons of least square means were done using PDIFF, and differences were significant at *P* < 0.05. The statistical model used is as follows:

(1) $$\begin{array}{c} Y_{ijkl}=\mu +\alpha_{i}+\beta_{j}+\alpha \beta \gamma_{ijk}+e_{ijkl}, \end{array}$$

where *Y*_*ijkl*_ is a dependent variable (fatty acid profile, lipid oxidation, and proximate composition), *μ* is the overall mean, *α*_*i*_ is the effect of fat (0%, 10%, and 15%), *β*_*j*_ is the effect of *Moringa oleifera* leaf meal (0%, 0.25%, and 0.5% MOLM), *αβγ*_*ijk*_ is the interaction (between fat and MOLM at different levels of inclusion), and *e*_*ijkl*_ is random error.

## 3. Results

### 3.1. Proximate Composition of Droëwors

Results of protein, moisture, fat, and ash for each treatment are presented in Table 2. The results showed that droëwors moisture content was within 10.79 ± 1.83 and 16.93 ± 1.29%. The protein and fat contents were within 45.80 ± 1.65 and 67.56 ± 2.33% and 14.37 ± 2.15 and 26.13 ± 2.15%, respectively. The ash content was within 10.66 ± 0.65 and 14.92 ± 0.65. T6 had a higher (*P* < 0.05) ash content than T4 and T5. There were no significant differences in moisture content across treatments. T1-T3 had higher protein (63.25 ± 1.65 to 67.56 ± 2.33%) than T4-T6 (58.30 ± 1.65 to 59.40 ± 1.65%) and T7-T9 (45.80 ± 1.65 to 54.16 ± 1.65%). T6 had significantly higher ash content (14.92 ± 0.65%) than T4 (11.36 ± 0.65%) and T5 (12.45 ± 0.65%). However, this increasing trend was variable or insignificant across other treatments with the same fat inclusion levels and increasing MOLM levels.

### 3.2. Fatty Acid Profile of Chicken Droëwors

Table 3 shows the fatty acid profiles of chicken droëwors with varying levels of MOLM and fat. The results showed that C18:1c9 (oleic: 30.95 to 32.65%) acid had the highest concentration, followed by C18:2c9, 12 (n-6) (linoleic: 28.84 to 30.30%), C16:0 (palmitic: 19.91 to 22.01%), and C18:0 (stearic: 6.23 to 7.31) acid. Droëwors with 0% fat and 0.25-0.5% MOLM added (T2, T3) had significantly lower n6/n3 ratios than droëwors with 10% fat (T4, T6) and 15% fat (T7-T9). From a health perspective, a lower n6:n3 is more favorable; however, the n6:n3 ratio in all treatments exceeded the recommended range of 3 : 1–1 : 1. The highest saturated fatty acid (SFA), monosaturated fatty acid (MUFA), and highest polyunsaturated fatty acid (PUFA) were obtained in T7, T6, and T9, respectively (Table 4).

### 3.3. Lipid Oxidation

Table 5 shows the lipid oxidation (TBARS) values. At the end of drying (72 h), droëwors with higher fat levels and 0.5% MO added (T6, T9) had lower (*P* < 0.05) TBARS than droëwors with 0% (T4, T7) and 0.25% (T5, T8). After 168 h of storage, TBARS in T1 (0.12 ± 0.01 mg MDA/kg), T4 (0.13 ± 0.01 mg MDA/kg), and T7 (0.10 ± 0.00 mg MDA/kg) were significantly higher than T2 (0.05 ± 0.03 mg MDA/kg), T3 (0.01 ± 0.00 mg MDA/kg), T6 (0.04 ± 0.01 mg MDA/kg), T8 (0.03 ± 0.02 mg MDA/kg), and T9 (0.05 ± 0.01 mg MDA/kg).

## 4. Discussion

### 4.1. Proximate Composition of Chicken Droëwors

Raw chicken has an average moisture content of 75% [20]. The present results indicated that moisture content was lower after drying, ranging from an average of 16.93 ± 1.29% to 10.79 ± 1.83%. This was anticipated since the droëwors lose up to 45% of their original mass due to moisture loss during drying. Similar studies reported identical results, where moisture content decreased after drying [21]. The moisture content range of chicken droëwors in this study (10.79-16.93%) was lower than the reported moisture content of common droëwors made from beef (19.9-31.7 g/100 g), game meat (17.6-35.3 g/100 g), and ostrich (27.3-31.5 g/100 g) [3]. In the current results, the MOLM treatments had similar moisture levels. A similar study on the oxidative stability of blesbok, springbok, and fallow deer droëwors when rooibos extract was added found that treatments with rooibos extract did not influence the amount of mass loss [1]. As expected, the fat level was significantly higher when more fat was added. The proximate composition of droëwors prepared with lean chicken meat was less fatty with substantially higher protein. Other similar studies have reported that droëwors made with lower fat inclusion levels resulted in lower moisture content after drying and higher protein and ash content [21]. Droëwors with higher fat inclusion levels have a higher moisture content because fat has a barrier effect on moisture transfer during drying [22]. The range of fat in chicken droëwors in this study (14.37-26.13%) was lower than the fat content of common droëwors made from beef (25.0-40.3 g/100 g), game meat (16.8-47.0 g/100 g), and ostrich (23.5-28.8 g/100 g). The lower fat content of chicken droëwors could be appealing to health-conscious consumers. In the treatments with 10% fat added, droëwors with 0.5% MOLM added (T6) had higher (*P* < 0.05) ash content than the treatments with 0 and 0.25% MOLM. This may be attributed to additional minerals in the MOLM, as they are reportedly a rich source of vitamins and minerals [10]. Higher levels of minerals could also be due to spices' addition [23]. However, in this study, the trend of increasing ash content with increasing MOLM level was inconsistent and not always significant across treatments.

### 4.2. Fatty Acid Profile of Chicken Droëwors

Chicken droëwors in the present study had high percentages of palmitic acid, stearic acid, linoleic acid, and oleic acid (Table 3). This finding could be attributed to the fact that chicken meat has a favorable fatty acid profile high in unsaturated fatty acids. Similar results were reported when rooibos extract was added to fallow deer, springbok, and blesbok droëwors [24]. Differences in the fatty acid profile in T1 and T9 could be presumed to be due to the difference in a specific treatment's fat and MOLM content. T9 (15% fat, 0.5% MOLM) had significantly higher PUFAs than T1 (0% fat, 0% MOLM) and T4 (10% fat, 0% MOLM). The difference is largely attributed to the higher level of fat inclusion as chicken fat is high in PUFAs, as reported elsewhere by Del Puerto et al. [2]. The increasing trend of PUFA content could be attributed to the increasing levels of MOLM, as more than half of the fatty acids in MOLM are unsaturated, as reported by Moyo et al. [25].

The results of the present study support that the chicken droëwors produced in the present study are suitable for consumer health. The literature further indicates that an increase in PUFA content plays an essential role in human health. Furthermore, high PUFA content confers economic benefits due to the high antioxidant content in MOLM on inhibiting lipid oxidation inhibition in chicken droëwors, as reported by Prisacaru [26]. The droëwors samples had comparatively higher PUFA (33.20 to 34.83%) concentrations than SFA (26.85 to 27.96%). Therefore, oxidation was expected to happen in the final product because of PUFA high levels, causing faster oxidation, as reported by Verardo et al. [27]. However, the presence of MOLM in the product delayed oxidation, as reported by Falowo et al. [28].

### 4.3. Lipid Oxidation

Before drying, TBARS values were similar (*P* > 0.05) across all treatments. The inclusion of 0.25% and 0.5% of MOLM caused a reduction (*P* < 0.05) in lipid oxidation of T6 and T9 after drying and 168 h of storage. The TBARS may have reduced due to the inhibition of lipid oxidation by MOLM. *Moringa oleifera* contains polyphenols with antioxidant effects. Plant phenolic compounds contribute to their antioxidative capacity by free radicals' scavenging capability, as reported by Falowo et al. [28, 29]. Literature reports that the antioxidant activity of polyphenols is mainly derived from their redox properties, which promote the absorption and neutralization of free radicals, decomposing peroxides, and quenching singlet oxygen [30]. These results are similar to the results of the study which found that the inclusion of 0.50%, 0.75%, and 1.00% of *Moringa oleifera* in the chicken sausages resulted in lower TBARS [31]. In the current study, low TBARS were in treatment groups T2, T3, T5, T6, T8, and T9, where 0.5% and 0.25% MOLM were added. The outcome of the TBARS is further corroborated by the finding of the effect of MOLM powder in another study on drying kinetics, *α*-tocopherol, *β*-carotene, ferric reducing antioxidant power, physicochemical properties, and lipid oxidation of dry pork sausages at processing and storage [18]. The study also reported significantly lower TBARS in pork droëwors with 0.75% *Moringa oleifera* leaf powder during drying and up to 10 days of storage. Like chicken droëwors, pork droëwors are reportedly more susceptible to lipid oxidation due to their high PUFA content [3]. Also, lower TBARS were reported with MOLM inclusion in a study conducted on ground pork [32]. Another study reported a positive correlation between MOLM and reduced lipid oxidation in meat products [33]. The positive correlation can be attributed to its antioxidant activity.

Higher TBARS were documented in T1, T4, and T7. The high level of TBARS could be associated with the noninclusion of MOLM in the previously mentioned treatments. Similar results were reported in pork droëwors in a similar study where no *Moringa oleifera* and no other antioxidants were added [18]. The results indicate that the inclusion of MOLM in the present research delayed lipid oxidation in chicken droëwors.

## 5. Conclusion

The present study showed that adding 0.5% Moringa oleifera leaf meal (MOLM) to chicken droëwors decreased lipid oxidation after 72 h of drying and 0.25-0.5% MOLM reduced lipid oxidation during 168 h of storage. There were high levels of stearic, palmitic, linoleic, and oleic fatty acids in chicken droëwors. With the predisposition of unsaturated fatty acids to lipid oxidation, the incorporation of MOLM as a natural antioxidant indicates that chicken meat could successfully be used to provide an alternative droëwors option for consumers.

## Figures and Tables

**Table 1:** Droëwors treatments prepared with different combinations of fat and MOLM.

Treatments	Fat inclusion level (%)	MOLM inclusion level (%)
T1	0	0
T2	0	0.25
T3	0	0.5
T4	10	0
T5	10	0.25
T6	10	0.5
T7	15	0
T8	15	0.25
T9	15	0.5

MOLM: *Moringa oleifera* leaf meal.

**Table 2:** Proximate composition of chicken droëwors produced with graded *Moringa oleifera* leaf meal and fat levels (least square means ± SE).

Treatments									
Components (%)	T1	T2	T3	T4	T5	T6	T7	T8	T9
Ash	13.95^ab^ ± 0.93	14.17^a^ ± 0.65	13.08^ab^ ± 0.05	11.36^b^ ± 0.65	12.45^b^ ± 0.65	14.92^a^ ± 0.65	14.69^a^ ± 0.65	10.66^b^ ± 0.65	13.51^ab^ ± 0.65
Fat	15.28^b^ ± 3.04	14.69^b^ ± 2.15	14.37^b^ ± 2.15	25.39^a^ ± 2.15	23.28^a^ ± 2.15	26.13^a^ ± 2.15	23.67^a^ ± 2.15	24.10^a^ ± 2.15	21.93^a^ ± 2.15
Moisture	10.79^a^ ± 1.83	13.19^a^ ± 1.29	13.90^a^ ± 1.29	15.45^a^ ± 1.29	15.64^a^ ± 1.29	15.50^a^ ± 1.29	14.82^a^ ± 1.29	16.93^a^ ± 1.29	14.99^a^ ± 1.29
Protein	67.56^a^ ± 2.33	63.25^a^ ± 1.65	67.27^ac^ ± 1.65	58.30^b^ ± 1.65	58.99^b^ ± 1.65	59.40^b^ ± 1.65	53.69^b^ ± 1.65	54.16^b^ ± 1.65	45.80^c^ ± 1.65

^ab^Means in the same row with different superscripts are significantly different (*P* < 0.05); T1 = 0% fat, 0 *Moringa oleifera* leaf meal (MOLM); T2 = 0% fat, 0.25% MOLM; T3 = 0% fat, 0.5% MOLM; T4 = 10% fat, 0% MOLM; T5 = 10% fat, 0.25% MOLM; T6 = 10% fat, 0.5% MOLM; T7 = 15% fat, 0% MOLM; T8 = 15% fat, 0.25% MOLM; T9 = 15% fat, 0.5% MOLM.

**Table 3:** Fatty acid composition (%) of chicken droëwors produced with graded *Moringa oleifera* leaf meal and fat levels.

Treatments									
Fatty acid (% total fatty acids)	T1	T2	T3	T4	T5	T6	T7	T8	T9
C14:0	0.43^b^ ± 0.12	0.465^ab^ ± 0.12	0.470^a^ ± 0.12	0.44^ab^ ± 0.12	0.46^ab^ ± 0.12	0.46^ab^ ± 0.12	0.46^ab^ ± 0.12	0.48^a^ ± 0.12	0.44^ab^ ± 0.12
C15:0	0.03^C^ ± 0.01	0.06^ab^ ± 0.01	0.07^a^ ± 0.01	0.04^bc^ ± 0.01	0.05^abc^ ± 0.01	0.04^bc^ ± 0.01	0.03^c^ ± 0.01	0.04^bc^ ± 0.01	0.04^bc^ ± 0.01
C16:0	19.91^C^ ± 0.42	21.00^abc^ ± 0.42	21.44^ab^ ± 0.42	20.34^bc^ ± 0.42	20.78^abc^ ± 0.42	20.48^bc^ ± 0.42	22.01^a^ ± 0.42	20.96^abc^ ± 0.42	20.84^abc^ ± 0.42
C17:0	0.11^a^ ± 0.01	0.12^a^ ± 0.01	0.12^a^ ± 0.01	0.11^a^ ± 0.01	0.11^a^ ± 0.01	0.12^a^ ± 0.01	0.13^a^ ± 0.01	0.12^a^ ± 0.01	0.12^a^ ± 0.01
C18:0	6.23^a^ ± 0.41	6.41^a^ ± 0.41	6.93^a^ ± 0.41	6.54^a^ ± 0.41	6.40^a^ ± 0.41	6.35^a^ ± 0.41	7.31^a^ ± 0.41	6.46^a^ ± 0.41	6.42^a^ ± 0.41
C20:0	0.06^a^ ± 0.00	0.07^a^ ± 0.00	0.07^a^ ± 0.00	0.07^a^ ± 0.00	0.07^a^ ± 0.00	0.07^a^ ± 0.00	0.07^a^ ± 0.00	0.07^a^ ± 0.00	0.07^a^ ± 0.00
C24:0	0.00^b^ ± 0.01	0.02^a^ ± 0.01	0.02^a^ ± 0.01	0.00^b^ ± 0.01	0.01^b^ ± 0.01	0.00^b^ ± 0.01	0.00^b^ ± 0.01	0.00^b^ ± 0.01	0.00^b^ ± 0.00
Monosaturated fatty acid									
C14:1c9	0.05^a^ ± 0.01	0.06^a^ ± 0.01	0.05^a^ ± 0.01	0.05^a^ ± 0.01	0.05^a^ ± 0.01	0.05^a^ ± 0.01	0.04^a^ ± 0.01	0.05^a^ ± 0.01	0.04^a^ ± 0.01
C16:1c9	3.74^a^ ± 0.14	3.77^a^ ± 0.14	3.41^a^ ± 0.14	3.38^a^ ± 0.14	3.47^a^ ± 0.14	3.45^a^ ± 0.14	3.16^a^ ± 0.14	3.35^a^ ± 0.14	3.45^a^ ± 0.14
C18:1 t9	0.02^a^ ± 0.00	0.02^ab^ ± 0.00	0.01^bc^ ± 0.00	0.01^bc^ ± 0.00	0.01^bc^ ± 0.00	0.01^bc^ ± 0.00	0.01^bc^ ± 0.00	0.01^bc^ ± 0.00	0.01^bc^ ± 0.00
C18:1c9	31.38^a^ ± 0.61	31.46^a^ ± 0.61	31.24^a^ ± 0.61	31.93^a^ ± 0.61	32.14^a^ ± 0.61	32.65^a^ ± 0.61	30.95^a^ ± 0.61	32.07^a^ ± 0.61	32.33^a^ ± 0.61
C18:1c7	2.93^ab^ ± 0.03	2.96^a^ ± 0.03	2.86^bc^ ± 0.03	2.91^ab^ ± 0.03	2.89^ab^ ± 0.03	2.90^ab^ ± 0.03	2.77^c^ ± 0.03	2.86^abc^ ± 0.03	2.89^ab^ ± 0.03
C18:2c9, 12 (n-6)	30.30^a^ ± 0.33	28.83^bc^ ± 0.33	28.83^bc^ ± 0.33	30.06^ab^ ± 0.33	29.56^abc^ ± 0.33	29.74^abc^ ± 0.33	28.84^c^ ± 0.33	29.80^abc^ ± 0.3	29.59^abc^ ± 0.33
C18:3c6, 9, 12 (n-6)	0.15^a^ ± 0.00	0.14^ab^ ± 0.00	0.13^b^ ± 0.00	0.15^a^ ± 0.00	0.14^ab^ ± 0.00	0.15^a^ ± 0.00	0.14^ab^ ± 0.00	0.15^a^ ± 0.00	0.15^a^ ± 0.00
C18:3c9, 12, 15 (n-3)	2.32^ab^ ± 0.05	2.41^a^ ± 0.05	2.38^a^ ± 0.05	2.36^ab^ ± 0.05	2.37^ab^ ± 0.05	2.32^ab^ ± 0.05	2.20^b^ ± 0.05	2.29^ab^ ± 0.05	2.33^ab^ ± 0.05
C22:1c13	0.23^a^ ± 0.02	0.20^ab^ ± 0.02	0.21^ab^ ± 0.02	0.11^ab^ ± 0.02	0.18^ab^ ± 0.02	0.16^b^ ± 0.02	0.19^ab^ ± 0.02	0.16^b^ ± 0.02	0.17^b^ ± 0.02
C20:4c5, 8, 11, 14 (n-6)	1.58^a^ ± 0.22	1.36^ab^ ± 0.22	1.31^ab^ ± 0.22	1.12^ab^ ± 0.22	1.04^ab^ ± 0.22	0.82^b^ ± 0.22	1.25^ab^ ± 0.22	0.88^ab^ ± 0.22	0.86^b^ ± 0.22
C20:5c5, 8, 11, 14, 17 (n-3)	0.02^a^ ± 0.00	0.02^a^ ± 0.00	0.02^a^ ± 0.00	0.00^b^ ± 0.00	0.00^b^ ± 0.00	0.01^b^ ± 0.00	0.00^b^ ± 0.00	0.00^b^ ± 0.00	0.00^b^ ± 0.00
Polyunsaturated fatty acid									
C20:2c11, 14 (n-)	0.18^a^ ± 0.01	0.19^a^ ± 0.01	0.19^a^ ± 0.01	0.16^a^ ± 0.01	0.17^a^ ± 0.01	0.15^a^ ± 0.01	0.15^a^ ± 0.01	0.15^a^ ± 0.01	0.15^a^ ± 0.01
C20:3c8, 11, 14 (n-6)	0.00^a^ ± 0.01	0.02^a^ ± 0.01	0.02^a^ ± 0.01	0.01^a^ ± 0.01	0.02^a^ ± 0.01	0.00^a^ ± 0.01	0.01^a^ ± 0.01	0.00^a^ ± 0.01	0.01^a^ ± 0.01
C22:5c7, 10, 13, 16, 19 (n-3)	0.18^a^ ± 0.03	0.14^a^ ± 0.03	0.14^a^ ± 0.03	0.16^a^ ± 0.03	0.10^a^ ± 0.03	0.09^a^ ± 0.03	0.12^a^ ± 0.03	0.08^a^ ± 0.03	0.09^a^ ± 0.03
C22:6c4, 7, 10, 13, 16, 19 (n-3)	0.09^a^ ± 0.02	0.07^a^ ± 0.02	0.06^ab^ ± 0.02	0.05^ab^ ± 0.02	0.02^b^ ± 0.02	0.02^b^ ± 0.02	0.06^ab^ ± 0.02	0.03^b^ ± 0.02	0.02^b^ ± 0.02

^abcd^Means in the same row with different superscripts are significantly different (*P* < 0.05), means in the same row with the same superscripts are not significant (*P* > 0.05); T1 = 0% fat, 0 *Moringa oleifera* leaf meal (MOLM); T2 = 0% fat, 0.25% MOLM; T3 = 0% fat, 0.5% MOLM; T4 = 10% fat, 0% MOLM; T5 = 10% fat, 0.25% MOLM; T6 = 10% fat, 0.5% MOLM; T7 = 15% fat, 0% MOLM; T8 = 15% fat, 0.25% MOLM; T9 = 15% fat, 0.5% MOLM.

**Table 4:** Fatty acid categories of chicken droëwors produced with graded *Moringa oleifera* leaf meal and fat levels (least square means ± SE).

Treatment									
Fatty acid	T1	T2	T3	T4	T5	T6	T7	T8	T9
SFA	26.85^b^ ± 0.83	28.23^ab^ ± 0.83	29.11^ab^ ± 0.83	27.53^ab^ ± 0.83	27.87^ab^ ± 0.83	27.51^ab^ ± 0.83	30.13^a^ ± 0.83	28.13^ab^ ± 0.83	27.96^ab^ ± 0.83
MUFA	38.34^a^ ± 0.72	38.45^a^ ± 0.72	37.78^a^ ± 0.72	38.47^a^ ± 0.72	38.73^a^ ± 0.72	39.22^a^ ± 0.72	37.11^a^ ± 0.72	38.49^a^ ± 0.72	38.88^a^ ± 0.72
PUFA	34.83^a^ ± 0.19	35.32^c^ ± 0.19	34.90^c^ ± 0.19	34.01^b^ ± 0.19	35.41^bc^ ± 0.19	33.29^c^ ± 0.19	32.76^c^ ± 0.19	36.38^bc^ ± 0.19	37.20^c^ ± 0.19
Omega-6	32.22^a^ ± 0.17	30.68^cd^ ± 0.17	30.55^cd^ ± 0.17	31.49^b^ ± 0.17	30.92^cd^ ± 0.17	30.86^cd^ ± 0.17	30.39^d^ ± 0.17	30.97^bc^ ± 0.17	30.75^cd^ ± 0.17
Omega-3	2.61^a^ ± 0.05	2.65^a^ ± 0.05	2.51^ab^ ± 0.05	2.52^abc^ ± 0.05	2.49^abc^ ± 0.05	2.43^c^ ± 0.05	2.38^c^ ± 0.05	2.41^c^ ± 0.05	2.45^bc^ ± 0.05
PUFA/SFA	1.30^a^ ± 0.04	1.18^bc^ ± 0.04	1.14^bc^ ± 0.04	1.24^ab^ ± 0.04	1.20^abc^ ± 0.04	1.21^abc^ ± 0.04	1.00^c^ ± 0.04	1.19^abc^ ± 0.04	1.19^abc^ ± 0.04
n-6/n-3	12.34^ab^ ± 0.21	11.62^c^ ± 0.21	11.71^bc^ ± 0.21	12.50^a^ ± 0.21	12.45^ab^ ± 0.21	12.71^a^ ± 0.21	12.79^a^ ± 0.21	12.84^a^ ± 0.21	12.57^a^ ± 0.21

^ab^Means in the same row with different superscripts are significantly different (*P* < 0.05); SFA = saturated fatty acid; MUFA = monosaturated fatty acid; PUFA = polyunsaturated fatty acid; PUFA/SFA = polyunsaturated fatty acids/saturated fatty acids; n-6/n-3 = omega-6 fatty acids/omega-3 fatty acids; T1 = 0% fat, 0 *Moringa oleifera* leaf meal (MOLM); T2 = 0% fat, 0.25% MOLM; T3 = 0% fat, 0.5% MOLM; T4 = 10% fat, 0% MOLM; T5 = 10% fat, 0.25% MOLM; T6 = 10% fat, 0.5% MOLM; T7 = 15% fat, 0% MOLM; T8 = 15% fat, 0.25% MOLM; T9 = 15% fat, 0.5% MOLM.

**Table 5:** Thiobarbituric acid reactive substances (TBARS) (mg MDA/kg meat) of chicken droëwors with added *Moringa oleifera* leaf meal (MOLM).

TBARS (mg MDA/kg meat)									
Time (hours)	T1	T2	T3	T4	T5	T6	T7	T8	T9
0	0.15^a^ ± 0.01	0.11^a^ ± 0.02	0.01^a^ ± 0.05	0.10^a^ ± 0.01	0.11^a^ ± 0.01	0.06^a^ ± 0.03	0.13^a^ ± 0.03	0.10^a^ ± 0.00	0.00^a^ ± 0.04
0.25	0.13^a^ ± 0.00	0.09^b^ ± 0.01	0.05^a^ ± 0.03	0.12^a^ ± 0.01	0.06^ab^ ± 0.03	0.05^a^ ± 0.01	0.10^a^ ± 0.01	0.07^b^ ± 0.01	0.05^a^ ± 0.02
0.5	0.16^a^ ± 0.01	0.08^ab^ ± 0.03	0.09^a^ ± 0.05	0.16^a^ ± 0.03	0.07^a^ ± 0.02	0.00^ab^ ± 0.03	0.16^a^ ± 0.02	0.02^ab^ ± 0.00	0.00^a^ ± 0.02
72	0.15^a^ ± 0.02	0.10^a^ ± 0.01	0.07^a^ ± 0.01	0.14^a^ ± 0.01	0.10^a^ ± 0.00	0.03^b^ ± 0.01	0.10^a^ ± 0.00	0.05^a^ ± 0.01	0.01^b^ ± 0.01
168	0.12^a^ ± 0.01	0.05^b^ ± 0.03	0.01^b^ ± 0.00	0.13^a^ ± 0.01	0.10^ab^ ± 0.05	0.04^b^ ± 0.01	0.10^a^ ± 0.00	0.03^b^ ± 0.02	0.05^b^ ± 0.01

^ab^Means in the same row with different superscripts are significantly different (*P* < 0.05); T1 = 0% fat, 0 *Moringa oleifera* leaf meal (MOLM); T2 = 0% fat, 0.25% MOLM; T3 = 0% fat, 0.5% MOLM; T4 = 10% fat, 0% MOLM; T5 = 10% fat, 0.25% MOLM; T6 = 10% fat, 0.5% MOLM; T7 = 15% fat, 0% MOLM; T8 = 15% fat, 0.25% MOLM; T9 = 15% fat, 0.5% MOLM.

## Data Availability

The experimental data used to support the findings of this study are included in the article.
